# Health information technology (IT) to improve the care of patients with chronic kidney disease (CKD)

**DOI:** 10.1186/1471-2369-15-7

**Published:** 2014-01-09

**Authors:** Clarissa J Diamantidis, Stefan Becker

**Affiliations:** 1 Division of Nephrology, University of Maryland School of Medicine, Baltimore, MD, USA; 2 Veterans Affairs Maryland Health Care System, Baltimore, MD, USA; 3 Department of Nephrology, University Duisburg-Essen, Essen, Germany

**Keywords:** Chronic kidney disease, Health information technology, Mobile health, Patient safety

## Abstract

Several reports show that patients with chronic disease who are empowered with information technology (IT) tools for monitoring, training and self-management have improved outcomes, however there are few such applications employed in kidney disease. This review explores the current and potential uses of health IT platforms to advance kidney disease care by offering innovative solutions to inform, engage and communicate with individuals with CKD.

## Introduction

More than 70 million individuals worldwide have chronic kidney disease (CKD) and according to estimates prevalence will further increase as will the already enormous impact on health systems resources related to CKD care [1,2]. Slowing disease progression and reducing adverse safety outcomes requires significant personal involvement of CKD patients to integrate complex recommendations that include medication adherence, lifestyle modification and nutritional adaptation. While studies indicate that higher specific knowledge is associated with improved self-management behavior in chronic disease, [3-6] individuals with CKD are often dissatisfied with their ability to communicate with their health care provider, and are frequently unaware of their CKD diagnosis or its implications [7-9]. As new technologies continue to provide consumers with access to a myriad of applications and portals to health information, a vast array of medical reference material is available to patients through the Internet and mobile applications, offering them a better understanding of their diseases and best practices. This review explores the current and potential uses of health IT platforms to advance kidney disease care by offering innovative solutions to inform, engage and communicate with individuals with CKD.

## Review

### The role of information technology in chronic disease

Use of the Internet and mobile devices to deliver health care is growing rapidly. It is estimated that 91% of the US population owns a mobile device, [10] and that 85% of US adults use the Internet or email, [11] and as a result electronic devices are increasingly used by both health care providers and patients as communication tools [12]. While individuals with chronic diseases are less likely than healthy adults to have Internet access (62% versus 81%, respectively), once online those with chronic diseases have a higher likelihood of using social media to share information [13]. Further, the availability of the Mobile Web is allowing individuals who may not have broadband capabilities readily available in their homes, to access Internet content with just the click of a button or the swipe of a finger. In fact, it is estimated that 34% of cell Internet users go online mostly using their phones, [14] and that black and English-speaking Latinos are the most active users of the Mobile Web, [15] which has direct implications in CKD where ethnic and racial minorities are disproportionately affected and suffer worse outcomes than their white counterparts [16,17].

Although various interventions using the Internet and mobile phones have been developed across a spectrum of chronic illnesses with promising results, [18-23] there are few such applications employed in kidney disease. Individuals with CKD who are frequently older and of lower socioeconomic status and health literacy, are often not in the target markets of IT providers and vendors, and as a result, a large portion of the CKD population may be overlooked as health care technologies emerge. Compounding the slow adoption of health IT in CKD is the overall poor insight and lack of awareness among both patients and healthcare providers of a CKD diagnosis, and the likelihood of prioritization of other chronic diseases such as diabetes and heart disease with less emphasis placed on kidney dysfunction in the overall care of such a highly comorbid individual. Nonetheless, providers using mobile technology to connect with their chronic disease patients improve overall patient-provider communication, strengthen patient autonomy, and empower patients to tackle daily health issues by becoming active participants in their own care, all of which are directly pertinent to the optimal care of the CKD population [21,24-28]. General themes among studies on the uses of health IT in chronic disease include prevention, compliance, surveillance, and disease management [29]. Examples of the latter themes include electronic self-documentation of blood sugars and weights in chronic conditions such as diabetes and congestive heart failure, respectively, which has been shown to be an effective strategy to promote patient self-activation [30,31]. Integrating novel health IT platforms into the routine care of individuals with CKD may enrich current strategies intended to improve the often poor outcomes of these patients.

### Web-based education

The Internet has become ubiquitous in society, with the availability of the World Wide Web on nearly every digital platform. Consequently, the bulk of digital educational materials available in kidney disease are available in some form on the Internet. The most well-established example of this in kidney disease is brought by the National Kidney Disease Education Program (NKDEP), which sponsors an initiative to promote kidney disease education via digital media. The NKDEP website contains several links to various kidney disease educational topics targeted for the patient, rather than the provider (http://www.nkdep.nih.gov). In addition, much of the web-based materials are available for download and print to be used as educational supplements during a provider-patient interaction that the patient can then take home and refer to as needed. The website content is directed at an elementary school level reading capability, and has been modified based on an iterative process of review [32]. This same iterative process was used in the development of the Safe Kidney Care Cohort study (SKC) website (http://www.safekidneycare.org), which provides information to patients, family members and providers, on topics relevant to patient safety in CKD [33]. After one year of follow-up, approximately 29% (n = 31) of SKC participants who were invited to visit the website (n = 108) did so, [34] which corroborates with other studies of website usage, revealing that approximately one third of study participants will visit a website when asked [35,36].

The Internet has also become a place where individuals can privately explore topics that may not be addressed in a typical office setting due to time constraints or uneasiness in speaking with providers, particularly for those who prefer time to absorb information at their own pace. Gordon et al. examined the quality of Hispanic-directed web-based educational materials for kidney donation and found significant heterogeneity in the content of the sites, and suggested a more comprehensive Hispanic-targeting educational platform would help increase knowledge regarding living kidney donation in this population [37]. Schatell examined the role of the Internet in promoting self-management in individuals on dialysis and those with pre-dialysis CKD, and proposed that the value of the Internet goes beyond the provision of static educational content; the Internet has become a resource for the development of social support systems for those affected by kidney disease [38]. For example, the National Kidney Foundation (NKF) has over 85,000 “likes” on Facebook (http://www.facebook.com/nationalkidneyfoundation) and over 9000 followers on Twitter (twitter.com/nkf) as of September 2013. While this is certainly nowhere near the estimated 26 million Americans affected by kidney disease, the effect of these social media outlets are far more widespread than most other kidney disease educational initiatives.

### Short message service (SMS) texting

SMS texting has been the most extensively studied health IT platform in chronic diseases, yet little work has been done to evaluate the potential benefit of this platform in individuals with CKD. In other conditions, texting has been used to provide appointment reminders,[39,40] help aid tobacco cessation, [26,41,42] improve physical activity and stimulate weight management efforts, [43-45] and improve diabetic control [46-49] among countless other applications. Studies from various clinical contexts in which text-messaging services were introduced, reported improved medication adherence [21,24-28,50]. While the mobile phone finds itself in the hands of individuals on all continents, investigators and health care providers have recognized an opportunity to reach individuals where perhaps vehicles, clinics or even computers cannot. SMS texting has become an integral part of “rural medicine”, examples of which include Lester et al’s study examining the effects of reminder texts on antiretroviral adherence in Kenya [51] and Asiimwe et al’s study evaluating the utility of an SMS text-based reporting system of malarial outbreaks in Uganda [52].

To some degree, kidney disease researchers are just now beginning to explore the utility of SMS text in the care of individuals with CKD. One co-author and colleagues examined a multi-platform medication inquiry system designed to provide information about the safety of drugs in pre-dialysis CKD, and found the SMS texting-based platform to be the least preferred by participants despite a comparable amount of technical errors noted on this platform when compared with the web-based or personal digital assistant (PDA)-based applications [53]. It is important to consider the implications of using an SMS text-based platform as a few characters must transmit large amount of meaning, and there is a greater potential for misunderstanding using this approach [54]. The overall acceptance of this type of application in a broader CKD population remains unclear, as does its utility in improving adherence or disease outcomes.

### Mobile apps

“Mobile Computing”, which is a broad term comprised of various forms of hardware (i.e. smartphones, tablet computers) is presently one of the most important technological trends, and offers promise for a “system solution” for patients with chronic disease. It allows the user to download mobile applications (“apps”) via the Internet, which can then be applied for various activities of daily life [55]. Worldwide there were almost 6 billion mobile phones being used in late 2011, more than one billion of these had broadband capabilities, and 43 billion mobile applications were downloaded in the 12 months ending September 2012 [56,57]. With the global prevalence of mobile technology, accessing health-related applications via mobile phone seems a logical approach to promote patient engagement [56]. In fact, according to a US study, 25% of smartphone users were already using such health applications and almost half of those asked would be interested in doing so [58].

To date, the majority of mobile apps available in kidney disease have focused on the provider or highly motivated patients. A query of “chronic kidney disease” is the Apple App Store™ will produce only a handful of relevant apps, among them Renal Trkrr (available for $7.99 in the App Store™), which is a comprehensive tool enabling individuals with CKD the opportunity to record their dietary intake, track their renal function (by user input), and communicate with their health care provider (by inputting provider email addresses). While the comprehensive nature of this tool is certainly admirable, the level of technical and clinical aptitude needed to store data, in addition to the relatively high cost, likely may make this novel application prohibitive to a population which is largely on the margin with regards to socioeconomic status and e-literacy. Further study is needed to determine effective disease-tailored mobile apps for the unique population with kidney disease.

### Barriers and considerations: lessons learned from the iNephro study

The iNephro study investigated how far a “native” smartphone application (“Medication Plan”) which allowed users the ability to maintain and alter personal drug therapy plans and document vital signs on their personal device, would be downloaded and used by general smartphone users [59]. Although there were more than 11000 app users within the first 15 months, the interest compared to apps targeting individuals with acute rather than chronic medical conditions has been reported to be much higher [56]. Most of the users seem to have been “early adopters” of a new service: middle-aged male, well-educated and derived from the relatively small number of daily taken pills - comparatively healthy [60]. This discrepancy or “digital divide” between age groups, sexes and education levels highlights the importance of an iterative development process in order to reach the needs of the intended audience, as the “digital divide” implies that certain parts of the population have better opportunities to benefit from modern technologies than others due to access, awareness, and a desire to adopt new technologies [61-63].

The iNephro study reported that the regular use of the application decreased considerably within the first 2 months and lasted for more than a year in only a few cases. This finding is consistent with other studies citing a high attrition rate for Internet interventions, [34,64] which may be a reflection of an early interest in the novelty of the application, with a decline in eagerness as the newness of the intervention wears off. At present there is no scientific data on why people stop using mobile health applications. In a commercial survey by the Consumer Health Information Corporation (CHIC) on the use of healthcare apps in 2011 users reported “not user friendly” and “found a better one” as the two most frequent reasons for no longer using an app (32% and 34% respectively). 26% of health apps were used only once after download and the dropout rate recorded in this survey was 74% (of 395 participants) by 10th use [65]. The fact that “Medication Plan” was not used permanently might therefore reflect deficits in usability and once again call attention to the necessity to continuously monitor consumer/patients’ demands. Others have found that most patients value interactive systems, i.e. devices that will give feedback - in this case on correct drug intake, a feature as yet not included in “Medication Plan” [66,67]. Also, patients seem to prefer to use mobile health (mHealth) in conjunction with visits to their doctors - another element which was not part of the iNephro study [66,67]. In essence, use of such an app can be seen as an expression of patients’ wishes to improve the effectiveness of their treatment by improving their adherence. The most favorable - if speculative - explanation for the decline in use of “Medication Plan” is that it may only have been used temporarily as a learning tool at least by some patients: once habituation to correct drug intake had been achieved, assistance was no longer regarded as necessary. Following this train of thought the fact that older individuals seemed to be using the app for a longer period than younger users may reflect a lesser slope in the learning curve of older users and the temporary adoption of the app as a companion tool.

### Perspective for mHealth

In the present climate it seems likely that health-related mobile computing applications will increasingly play a role for elderly and chronically ill users for a variety of reasons. Currently, most of the hardware and commercially successful applications are likely to be targeting younger (between 18 – 40 years of age) and healthier individuals, as they constitute the majority of contemporary smartphone users [55]. However, the young and middle-aged of today are the CKD patients of tomorrow. This cohort effect will alter the demographics of those who rely on information technology and who increasingly integrate the mobile Internet into their daily lives [56,64]. Future developments should take into consideration that in general, Internet use is inversely correlated with an increasing number of medical conditions and lack of financial resources [68]. Also, a decrease of associated costs of mobile devices, i.e. costs for smartphones and tablet computers, may be essential to mitigate the “digital divide”. From the iNephro study it is known that in many cases the choice of consumers to download the application seems to have been promoted by media recommendations [69]. This reflects the element of novelty in apps like “Medication Plan”. It is likely that some form of guidance and possibly coaching by the medical profession will - for the time being - be necessary to establish health-related apps as a fixture in patient care. In this context online-platforms like “Happtique”, which provides certification for mobile health app operability (http://www.happtique.com/platform/) may play an important role in streamlining the quality of mHealth materials for distribution to patients and healthcare professionals.

### Other health IT delivery methods

In addition to web-based and mobile phone-based applications, several other uses for health IT are currently being explored in kidney disease. One such is example is the use of Telehealth, which is a broad term referring to the use of a technology in health care delivery. Possibilities for Telehealth interventions are wide-ranging and include the delivery of telephone-based educational materials and prompts via landline, to video-conferenced clinical visits (also known as “Telenephrology”). Other health IT practices such as the use of interactive voice response system (IVRS)-based applications may have appeal in CKD, particularly as IVRS does not require a high degree of technical aptitude for correct usage and can be used from any type of phone. Further, the use of the personal health record (PHR) by patients to communicate with their healthcare provider, obtain lab results, and view appointment schedules, has become a widespread initiative by various agencies in an effort to streamline the delivery of care to patients [70,71], however the long-term effect of such initiatives has yet to be determined.

Finally personable wearable fitness devices or passive sensor technology are more and more deployed in patient care [72]. Traditionally, these have been used to measure vital signs such as heart rhythm, oxygen level, and blood pressure [73,74], however, an interesting approach for measuring kidney function is under development by the Fraunhofer Institute for Reliability and Microintegration (IZM), Berlin [75,76]. This technology would involve reduction in a fluorescent radiation as the kidney excretes an injected colorant, allowing for non-invasive monitoring of GFR [75]. In the future such a technology may allow continually and more accurate monitoring of the kidney function at a lesser cost.

## Conclusion

The possible uses of health IT as a method to engage individuals with CKD are wide-ranging. In a perfect CKD world, individuals would be able to view comprehensive therapy plans and medical records issued by physicians on their personal mobile devices, with assurance of confidentiality and ease of accessibility. Applications would be available on multiple platforms, created using an iterative development process, and tailored closely to the specific demands of the CKD community to improve the likelihood of acceptance as part of an often complicated day-to day routine. Simplicity would be a vital element to any CKD-directed health IT application, particularly when targeting users with low e-literacy. And while health IT can never replace the provider-patient relationship, it can be integrated to create more effective and efficient treatment strategies for individuals with CKD. The future is closer than you think.

## Abbreviations

CKD: Chronic kidney disease; Health IT: Health information technology; SKC: Safe kidney care; mHealth: Mobile health.

## Competing interests

The authors declare that they have no competing interests.

## Authors’ contributions

CD and SB contributed equally to the design and drafting of this review. Both authors read and approved the final manuscript.

## Authors’ information

CD is an Assistant Professor of Nephrology in the Division of Nephrology at the University of Maryland School of Medicine and has published work related to the use of health information technology in chronic kidney disease. SB is Oberarzt at the Department of Nephrology and head of the iNephro Initiative at University Duisburg-Essen, Essen, Germany.

## Pre-publication history

The pre-publication history for this paper can be accessed here:

http://www.biomedcentral.com/1471-2369/15/7/prepub
